# Deep learning-based phenotyping reclassifies combined hepatocellular-cholangiocarcinoma

**DOI:** 10.1038/s41467-023-43749-3

**Published:** 2023-12-14

**Authors:** Julien Calderaro, Narmin Ghaffari Laleh, Qinghe Zeng, Pascale Maille, Loetitia Favre, Anaïs Pujals, Christophe Klein, Céline Bazille, Lara R. Heij, Arnaud Uguen, Tom Luedde, Luca Di Tommaso, Aurélie Beaufrère, Augustin Chatain, Delphine Gastineau, Cong Trung Nguyen, Hiep Nguyen-Canh, Khuyen Nguyen Thi, Viviane Gnemmi, Rondell P. Graham, Frédéric Charlotte, Dominique Wendum, Mukul Vij, Daniela S. Allende, Federico Aucejo, Alba Diaz, Benjamin Rivière, Astrid Herrero, Katja Evert, Diego Francesco Calvisi, Jérémy Augustin, Wei Qiang Leow, Howard Ho Wai Leung, Emmanuel Boleslawski, Mohamed Rela, Arnaud François, Anthony Wing-Hung Cha, Alejandro Forner, Maria Reig, Manon Allaire, Olivier Scatton, Denis Chatelain, Camille Boulagnon-Rombi, Nathalie Sturm, Benjamin Menahem, Eric Frouin, David Tougeron, Christophe Tournigand, Emmanuelle Kempf, Haeryoung Kim, Massih Ningarhari, Sophie Michalak-Provost, Purva Gopal, Raffaele Brustia, Eric Vibert, Kornelius Schulze, Darius F. Rüther, Sören A. Weidemann, Rami Rhaiem, Jean-Michel Pawlotsky, Xuchen Zhang, Alain Luciani, Sébastien Mulé, Alexis Laurent, Giuliana Amaddeo, Hélène Regnault, Eleonora De Martin, Christine Sempoux, Pooja Navale, Maria Westerhoff, Regina Cheuk-Lam Lo, Jan Bednarsch, Annette Gouw, Catherine Guettier, Marie Lequoy, Kenichi Harada, Pimsiri Sripongpun, Poowadon Wetwittayaklang, Nicolas Loménie, Jarukit Tantipisit, Apichat Kaewdech, Jeanne Shen, Valérie Paradis, Stefano Caruso, Jakob Nikolas Kather

**Affiliations:** 1 Université Paris Est Créteil, INSERM, IMRB, F-94010 Créteil, France; 2 Assistance Publique-Hôpitaux de Paris, Henri Mondor-Albert Chenevier University Hospital, Department of Pathology, Créteil, France; 3 Inserm, U955, Team 18, Créteil, France; 4 European Reference Network (ERN) RARE-LIVER, Créteil, France; 5 Else Kroener Fresenius Center for Digital Health, Medical Faculty Carl Gustav Carus, Technical University Dresden, Dresden, Germany; 6 Department of Medicine III, University Hospital RWTH Aachen, RWTH Aachen university, Aachen, Germany; 7 Centre d’Histologie, d’Imagerie et de Cytométrie (CHIC), Centre de Recherche des Cordeliers, Paris, France; 8 Laboratoire d’Informatique Paris Descartes (LIPADE), Université Paris Cité, Paris, France; 9 INSERM, Sorbonne Université, Université Paris Cité, Paris, France; 10 Caen University Hospital, Department of Pathology, Caen, France; 11 Department of Surgery and Transplantation, University Hospital RWTH Aachen, Aachen, Germany; 12 Institute of Pathology, University Hospital RWTH Aachen, Aachen, Germany; 13 CHRU Brest, Department of Pathology, Brest, 29220 France; 14 Univ Brest, Inserm, CHU de Brest, LBAI, UMR1227 Brest, France; 15 Clinic for Gastroenterology, Hepatology and Infectious Diseases, University Hospital Düsseldorf, Medical Faculty of Heinrich Heine University Düsseldorf, Düsseldorf, Germany; 16 Department of Pathology, Humanitas University, Humanitas Clinical and Research Center, IRCCS, Rozzano, Milan, Italy; 17 Assistance Publique–Hôpitaux de Paris, Beaujon University Hospital, Department of Pathology, F-92110 Clichy, France; 18 Université de Paris, Inflammation Research Center, Inserm, U1149, CNRS, ERL8252, F-75018 Paris, France; 19 Department of Pathology, E Hospital, Hanoi Medical University, Hanoi, Vietnam; 20 Pathology Center, Bachmai Hospital, Hanoi, Vietnam; 21 Department of Human Pathology, Kanazawa University Graduate School of Medicine, Kanazawa, Japan; 22 Pathology and Molecular biology Center, National Cancer Hospital, Hanoi, Vietnam; 23 University Lille, UMR9020-U1277, Cancer Heterogeneity Plasticity and Resistance to Therapies, Lille, France; 24 CHU Lille, Institute of Pathology, Lille, France; 25 Department of Laboratory Medicine and Pathology, Mayo Clinic Rochester, Rochester, MN USA; 26 Assistance Publique-Hôpitaux de Paris, Pitié-Salpêtrière University Hospital, Department of Pathology, Paris, France; 27 Assistance Publique-Hôpitaux de Paris, Saint-Antoine University Hospital, Department of Pathology, Paris, France; 28 Department of Pathology, Dr Rela Institute and Medical Centre, Bharath Institute of Higher Education and Research, Chennai, India; 29 Department of Hepatobiliary Pathology, Cleveland Clinic Foundation, Cleveland, OH USA; 30 Robert J. Tomsich Pathology & Laboratory Medicine Institute, Cleveland Clinic, 9500 Euclid Avenue, L25, Cleveland, OH 44195 USA; 31 Department of Gastrointestinal and Hepatobiliary Surgery, Cleveland Clinic Foundation, Cleveland, OH USA; 32 Barcelona Clinic Liver Cancer (BCLC) Group, Department of Pathology, Hospital Clínic de Barcelona, Universitat de Barcelona, Barcelona, Spain; 33 Department of Pathology, Gui-de-Chauliac University Hospital, 80, avenue Augustin-Fliche, 34295 Montpellier, France; 34 Department of Digestive and Hepatobiliary Surgery, Gui-de-Chauliac University Hospital, 80, avenue Augustin-Fliche, 34295 Montpellier, France; 35 Institute of Pathology, University of Regensburg, Franz-Josef-Strauß-Allee 11, 93053 Regensburg, Germany; 36 Department of Anatomical Pathology, Singapore General Hospital, Singapore, Singapore; 37 Department of Anatomical and Cellular Pathology, The Chinese University of Hong Kong, Shatin, Hong Kong; 38 CHU Lille, Department of Digestive and Hepatobiliary Surgery, Lille, France; 39 Dr Rela Institute and Medical Centre, Bharath Institute of Higher Education and Research, Chennai, India; 40 Rouen University Hospital, Department of Pathology, Rouen, France; 41 Barcelona Clinic Liver Cancer (BCLC), Liver Unit, Hospital Clinic of Barcelona, IDIBAPS, CIBEREHD, Universidad de Barcelona, Barcelona, Spain; 42 Assistance Publique-Hôpitaux de Paris, Pitié-Salpêtrière University Hospital, Department of Hepatology, Paris, France; 43 Assistance Publique-Hôpitaux de Paris, Pitié-Salpêtrière University Hospital, Department of Digestive and Hepatobiliary Surgery, Paris, France; 44 Centre Hospitalier Universitaire d’Amiens, Département de Pathologie, Amiens, France; 45 Reims University Hospital, Department of Pathology, Reims, France; 46 Department of Pathology, University Hospital, Grenoble, France; 47 Translational Innovation in Medicine and Complexity, Centre National de la Recherche Scientifique UMR5525, La Tronche, France; 48 Caen University Hospital, Department of Digestive and Hepatobiliary Surgery, Caen, France; 49 Poitiers University Hospital, Department of Pathology, Poitiers, France; 50 LITEC, Université de Poitiers, Poitiers, France; 51 Poitiers University Hospital, Department of Hepatogastroenterology and Oncology, Poitiers, France; 52 Assistance Publique-Hôpitaux de Paris, Henri Mondor-Albert Chenevier University Hospital, Department of Medical Oncology, Créteil, France; 53 Department of Pathology, Seoul National University Hospital, Seoul National University College of Medicine, Seoul, Korea; 54 CHU Lille, Department of Hepatology, Lille, France; 55 Angers University Hospital, Department of Pathology, Angers, France; 56 Department of Pathology, UT Southwestern Medical Center, Dallas, TX USA; 57 Assistance Publique-Hôpitaux de Paris, Henri Mondor University Hospital, Department of Digestive and Hepatobiliary Surgery, Créteil, France; 58 Assistance Publique-Hôpitaux de Paris, Paul Brousse University Hospital, Department of Digestive and Hepatobiliary Surgery, Paris, France; 59 Department of Internal Medicine, University Medical Center Hamburg-Eppendorf, Hamburg, Germany; 60 Department of Pathology, University Medical Center Hamburg-Eppendorf, Hamburg, Germany; 61 Reims University Hospital, Department of Digestive and Hepatobiliary Surgery, Reims, France; 62 Department of Pathology, Yale University School of Medicine, New Haven, CT USA; 63 Assistance Publique-Hôpitaux de Paris, Henri Mondor University Hospital, Department of Medical Imaging, Créteil, France; 64 Assistance Publique-Hôpitaux de Paris, Henri Mondor University Hospital, Department of Hepatology, Créteil, France; 65 Assistance Publique-Hôpitaux de Paris, Paul Brousse University Hospital, Department of Hepatology, Paris, France; 66 Institute of Pathology, Lausanne University Hospital (CHUV), University of Lausanne (UNIL), Lausanne, Switzerland; 67 Department of Pathology and Immunology, Washington University in St. Louis, St. Louis, MO 63110 USA; 68 Department of Pathology University of Michigan, Ann Arbor, MI USA; 69 Department of Pathology, The University of Hong Kong, Pok Fu Lam, Hong Kong, China; 70 State Key Laboratory of Liver Research, (The University of Hong Kong), Pok Fu Lam, Hong Kong, China; 71 Department of Pathology and Medical Biology, University Medical Center Groningen, Groningen, the Netherlands; 72 Assistance Publique-Hôpitaux de Paris, Paul Brousse University Hospital, Department of Pathology, Villejuif, France; 73 Assistance Publique-Hôpitaux de Paris, Saint Antoine University Hospital, Department of Hepatology, Paris, France; 74 Gastroenterology and Hepatology Unit, Division of Internal Medicine, Faculty of Medicine, Prince of Songkla University, Hat Yai, Thailand; 75 Prince of Songkla University, Department of Pathology, Hat Yai, Thailand; 76 Center for Artificial Intelligence in Medicine and Imaging, Stanford University, 1701 Page Mill Road, Palo Alto, CA 94304 USA; 77 Department of Pathology, Stanford University School of Medicine, 300 Pasteur Drive, Stanford, CA 94305 USA; 78 Medical Oncology, National Center for Tumor Diseases (NCT), University Hospital Heidelberg, Heidelberg, Germany

**Keywords:** Liver cancer, Tumour heterogeneity, Biomedical engineering, Machine learning, Pathology

## Abstract

Primary liver cancer arises either from hepatocytic or biliary lineage cells, giving rise to hepatocellular carcinoma (HCC) or intrahepatic cholangiocarcinoma (ICCA). Combined hepatocellular- cholangiocarcinomas (cHCC-CCA) exhibit equivocal or mixed features of both, causing diagnostic uncertainty and difficulty in determining proper management. Here, we perform a comprehensive deep learning-based phenotyping of multiple cohorts of patients. We show that deep learning can reproduce the diagnosis of HCC vs. CCA with a high performance. We analyze a series of 405 cHCC-CCA patients and demonstrate that the model can reclassify the tumors as HCC or ICCA, and that the predictions are consistent with clinical outcomes, genetic alterations and in situ spatial gene expression profiling. This type of approach could improve treatment decisions and ultimately clinical outcome for patients with rare and biphenotypic cancers such as cHCC-CCA.

## Abstract (web summary)

Combined hepatocellular-cholangiocarcinomas (cHCC-CCA) are challenging to diagnose, as they exhibit features of hepatocellular carcinoma (HCC) and intrahepatic cholangiocarcinoma (ICCA). Here, the authors use deep learning to re-classify cHCC-CCA tumours into HCC or ICCA based on histopathology images.

## Introduction

Primary liver cancer is the fourth leading cause of cancer-related death worldwide and an increasing public health problem^1^. The two most common types of primary liver cancer are hepatocellular carcinoma (HCC), which derives from hepatocytes, and intrahepatic cholangiocarcinoma (ICCA), which is thought to originate from biliary epithelial cells^1^. These two entities represent the two ends of the primary liver tumor spectrum and have completely different risk factors, clinical outcomes, treatment strategies and genetic/molecular features^1,2^.

Combined hepatocellular-cholangiocarcinoma (cHCC-CCA) is a rare variant of liver cancer which can present as a mixture or a coexistence of tumor tissue with hepatocellular and biliary morphological differentiation^3^. Most cases, however, display equivocal features that cannot be easily classified as either HCC or ICCA. This explains why the diagnosis is often very difficult for pathologists. Clinical management of patients with cHCC-CCA is also highly challenging, and, due to the rarity of this cancer, there are no consensus guidelines. Treatment strategies are usually extrapolated from HCC and ICCA, but the regulatory approval of modern therapies is usually restricted to “pure” HCCs or ICCAs. As a result, patients with cHCC-CCA often do not respond well to therapies and have detrimental clinical outcomes^3^. Interestingly, several studies showed that cHCC-CCA displays overlapping genetic alterations and gene expression profiles with those of HCC or ICCA, and it is debated whether cHCC-CCA represents a true molecular entity^3–5^. A recent study has suggested that cHCC-CCA arise from liver progenitor cells, and that its development is dependent on IL-6 trans-signaling^6^. Another hypothesis is that these tumors may indeed arise from the dedifferentiation or transdifferentiation of a preexisting conventional HCC or ICCA, but maintain a phylogenetic proximity to their ancestral differentiation^3^.

**Fig. 1:**
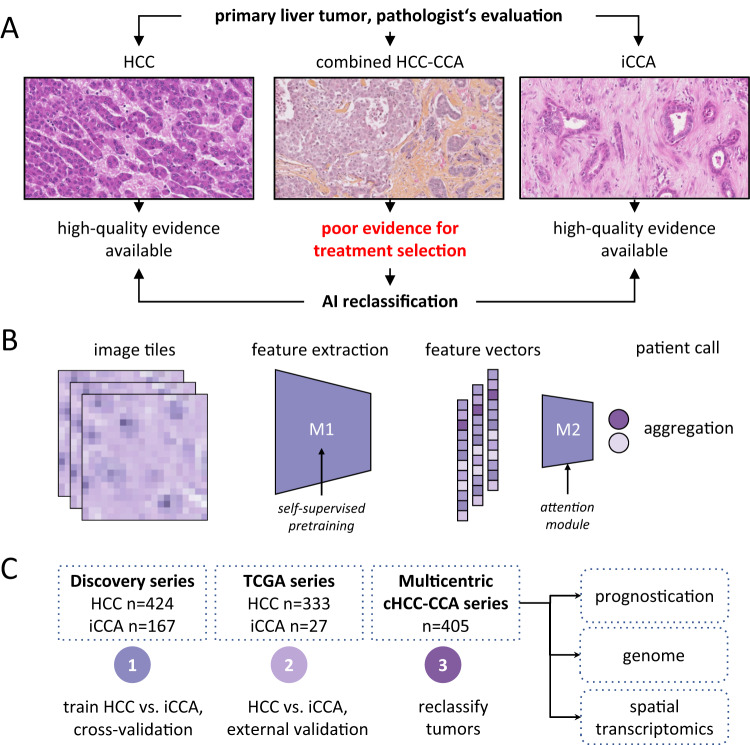
Deep Learning-based classification of HCC versus ICCA. **A** Clinical problem: combined HCC-CCA tumors are a diagnostic dilemma and only poor evidence is available to guide treatment in these patients. We hypothesize that an AI system can reclassify all cHCC-CCA cases into either category. **B** Technical approach: a two-step pipeline is used to transform image tiles into feature vectors by model 1 (M1), a pre-trained feature extraction model. A bag of feature vectors is subsequently aggregated into a call for a given patient by model 2 (M2), the aggregation model. **C** Experimental approach: a binary prediction model was trained to distinguish HCC from CCA and was evaluated via cross-validation and by external validation. Subsequently, the trained model was applied to a multicentric cohort of cHCC-CCA tumors and its predictions were comprehensively validated with a multimodal approach.

Artificial intelligence (AI) is widely used in pathology image analysis. We and others have applied AI to digitized whole slide images (WSI) of different cancers, including primary liver tumors, and showed that AI can extract clinically actionable information directly from routinely available tissue slides stained with hematoxylin and eosin (H&E)^7–10^. In this work, we aim to determine if AI allows the reclassification of cHCC-CCA as pure HCC or ICCA (Fig. 1A), and if this classification has both a clinical (in terms of prognostication) and a molecular (in terms of concordance with genetic defects and spatial molecular profiles) relevance.

## Results

### AI model performance in differentiating HCC and ICCA

**Fig. 2:**
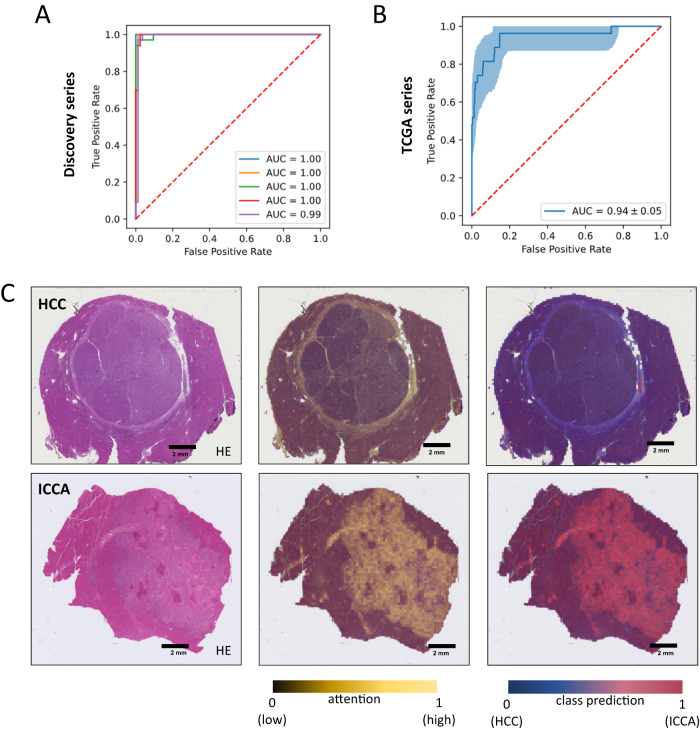
Development of a deep-learning model for HCC/ICCA classification. **A** Receiver operator curve (ROC) for the internal validation of binary classification of HCC and iCCA cases. **B** ROC curve for the external validation of binary classification (HCC vs. ICCA) task on TCGA dataset. The error band shows the 1000 fold bootstrapped 95% confidence interval. **C** H&E slide of two randomly selected cases for HCC and ICCA. Attention map of the model and the class prediction scores are used as explainability methods to check the capability of the trained model in detecting the correct features within the WSI. The class prediction heatmap is weighted by the attention. Source data are provided as a Source Data file. This analysis was repeated independently with similar results five times.

To investigate whether an AI model can re-classify cHCC-CCA tumors into “pure” HCC or ICCA categories, we trained an AI pipeline based on a self-supervised feature extractor^11^ with an attention-MIL aggregation model^12–14^ (Fig. 1B) to distinguish pure HCCs (785 WSIs from *n* = 424 patients) from pure ICCAs (239 WSIs from *n* = 167 patients) (Methods, Supplemental Tables 1 and 2). In this cohort (“Discovery cohort”, Fig. 1C), the model achieved a cross-validated area under the receiver operator characteristic curve (AUROC) of 0.99 [$\pm$0.01], corresponding to an almost perfect separability of the classes (Fig. 2A), reaching a sensitivity of 97.9% and specificity of 97.6%. As another piece of evidence for the plausibility of the model’s predictions, we subsequently evaluated the model on another patient cohort, the publicly available TCGA cohort, which was composed of *n* = 333 HCCs (TCGA-LIHC) and *n* = 27 ICCAs (TCGA-CHOL). The labels of the TCGA cohort were not seen by the model during training, however the training was exposed to some TCGA image data during self-supervised pretraining, which might affect an intermediary result but not the subsequent results. We found that the model reached an AUROC of 0.94 [$\pm$0.05], representing a very good generalizability to this additional dataset (Fig. 2B). Next, we asked which tissue structures were used by the model to make its prediction and found that the model placed a high attention to areas with an ICCA-like phenotype (glandular structures and fibrous stroma) (Supplemental Fig. 1). Together, these data show that the AI model can robustly distinguish pure HCC from pure ICCA tumors (Fig. 2C). We used this model as the starting point for our subsequent experiments.

### AI model application on cHCC-CCA samples

**Fig. 3:**
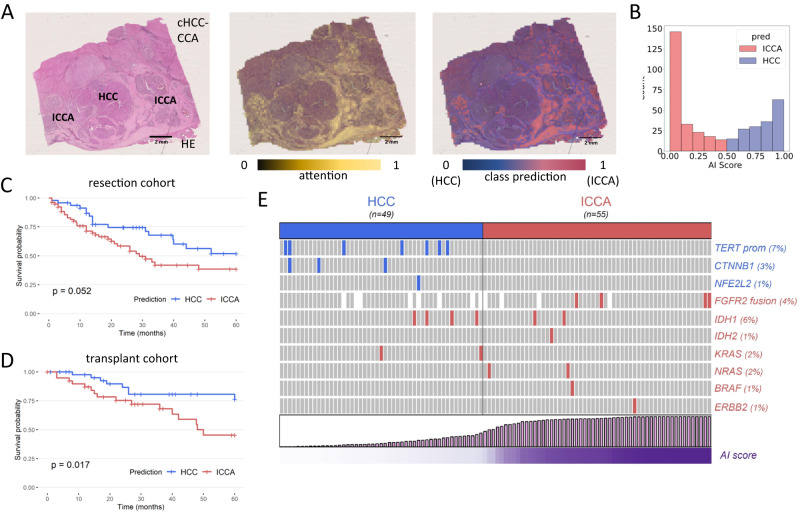
Reclassification of combined hepatocellular-cholangiocarcinomas. **A** Example of a HE slide of a cHCC-CCA and its associated attention and prediction heatmaps. This case features relatively distinct HCC and ICCA components, both of which are identified on the attention maps (attention is however higher in ICCA areas). The class predictions match the HCC and ICCA morphological contingents. This analysis was repeated independently with similar results five times. **B** Distribution of the raw outputs/predictions from the model: the scores follow a bimodal distribution, with a majority of cases peaking at a high HCC or ICCA. **C** Reclassification of cHCC-CCA as HCC or CCA has an impact on overall survival of patients treated by surgical resection. **D** Importantly, the prognosis value of the reclassification is validated in patients who underwent liver transplantation. **E** The model predictions match with the underlying alterations identified in cHCC-CCA (*p* = 0.0009, Fisher’s exact test). Statistical tests were two-sided and not adjusted for multiple testing. Source data are provided as a Source Data file.

Subsequently, we applied the trained model to a large multicentric cohort of cases which were initially diagnosed as cHCC-CCA (Supplemental Table 3). We investigated the spatial prediction maps and found that, generally, regions with HCC-like morphology were assigned a high “HCCness” by the model, while regions with ICCA-like morphology were assigned a high “ICCAness” by the model (Fig. 3A). For tumors with a significant proportion of equivocal or intermediate features, it was however much more difficult for pathologists to determine the morphology and proportion of areas with high “HCCness” or “ICCAness”. As region-specific predictions are not clinically actionable, we further investigated the patient-level prediction scores. We found that these scores followed a bimodal distribution, with a subset of cases peaking at a high HCC prediction and the remainder of the cases peaking at a high ICCA prediction (Fig. 3B). Importantly, there was no association between the predictions and the clinical centers managing the patients (*p* = 0.62). Together, these data show that the AI model can process tissue samples of cHCC-CCA cases and re-classify them as HCC or CCA. We then sought to determine if a simple pathological reclassification of cHCC-CCA tumors by microscopic examination was associated with AI predictions. To this aim, all cases were reviewed in a blinded way by an expert liver pathologist (JC) and cHCC-CCA were reclassified as HCC or ICCA according to the more abundant morphological component (Methods). Interestingly, only a slight concordance was observed between the pathological analysis and the model’s predictions (Cohen’s Kappa 0.19, Supplemental Fig. 2), indicating that the AI model does not simply assess the more abundant tissue component in the way a human pathologist would.

### Clinical outcomes based on AI-based reclassification

Next, we investigated the potential clinical and biological relevance of the AI-based reclassification of cHCC-ICCAs. One of the major differences between HCC and ICCA is their clinical outcome, with worse overall 5-year survival rates for patients with ICCA^3^. We thus aimed to determine if our reclassification had an impact on the prognosis. Indeed, patients with cHCC-CCA reclassified as ICCA had a shorter median survival (29 months) than patients with a tumor reclassified as HCC (median survival not reached, *p* = 0.052, hazard ratio = 1.76 95% CI 0.98-3.14, Fig. 3C and Supplemental Table 4). Survival prediction is particularly relevant in patients who receive a liver transplant, as donor organs should be prioritized for patients with a good prognosis. A diagnosis of cHCC-CCA is currently considered a contra-indication to this therapeutic modality which remains a curative option for patients with HCC. As observed for resection, patients with cHCC-CCA reclassified as HCC showed a prolonged 5-year overall survival (76.4% survival rate, median survival not reached), which is similar to that usually observed in patients with conventional HCC (Fig. 3D and Supplemental Table 5). Transplanted patients with an incidental diagnosis of cHCC-CCA at transplant, which were re-classified as ICCA by the AI model, had a poor median survival of 48 months and a 5-year overall survival of only 45.4% (hazard ratio = 2.69, 95% CI 1.15-6.31). As opposed to resected patients, the prognostic impact remained significant on multivariate analysis. This observation may be explained by the fact that the competing mortality from cirrhosis and the risk of tumor recurrence are minimized by liver transplantation, as the whole diseased liver is replaced by the intervention. We also observed differences in patient characteristics according to the 2 different therapeutic modalities: transplanted patients were more frequently male (*p* = 0.039), cirrhotic (*p* < 0.001), with a higher frequency of alcohol consumption (*p* < 0.001) and a lower rate of HBV infection (*p* = 0.004) (Supplemental Table 6).

We further aimed to determine if the conventional reclassification of cHCC-CCA (according to the more abundant contingent assessed by a blinded pathologist) yielded any prognostic value, but observed that it had not a significant impact on survival in either resected (*p* = 0.16) or transplanted (*p* = 0.32) patients (Supplemental Figs. 3 and 4). In order to assess the degree of inter-observer variability of this histological reclassification, slides were also reviewed by another pathologist. The overall agreement was only fair (Cohen’s Kappa of 0.37), supporting the use of a more standardized and reproducible system such as our model. Altogether, these data suggest that our AI-based reclassification of cHCC-CCA allows us to make more clinically relevant predictions about disease outcomes than a classical pathological assessment.

### AI-based reclassification and genomic alterations

We next investigated if the model’s predictions were concordant with known genetic differences of HCC and ICCA. We performed targeted next-generation sequencing with a panel that includes all major genes involved in HCC or ICCA development for *n* = 104 randomly selected cases. We identified several cases with alterations in *TERT* promoter, *CTNNB1* and *NFE2L2*, which typically occur in HCCs, and several cases with *FGFR2* fusions and *IDH1/2*, *KRAS*, *NRAS*, *BRAF* and *HER2* mutations, which typically occur in ICCAs. We found that all genetic alterations in HCC-specific genes occurred in the tumor subset which the AI model had re-classified as HCC (Fig. 3E). Eleven out of 16 genetic alterations which are typical for ICCA occurred in tumors that were re-classified as CCA. In other words, the AI predictions match the genomic alterations of cHCC-CCA (*p* = 0.0009 in Fisher’s exact test), suggesting that the model detects patterns directly linked to the genetic defects identified by genomic profiling of the tumor tissue.

### Spatial transcriptomics analysis and AI predictions

**Fig. 4:**
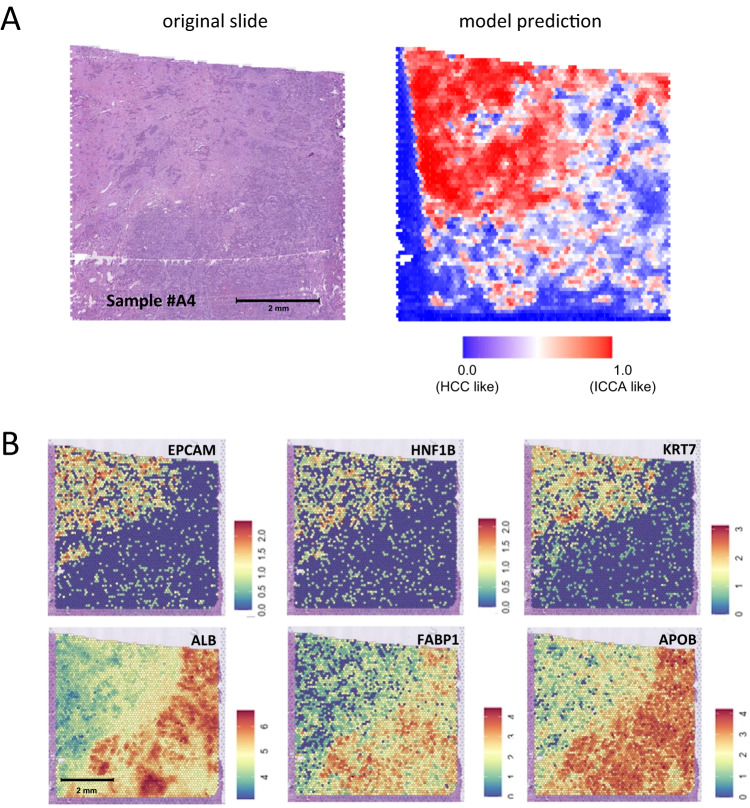
Combination of deep-learning heatmaps with spatial transcriptomics unravels the gene expression profile of areas that markedly impact the predictions. **A** Example of a case processed by spatial transcriptomics: the HE section and its corresponding prediction heat map are presented, with the upper left area being considered as ICCA-like. This analysis was repeated independently with similar results for two other cases, as mentioned in the section “Spatial Transcriptomics Analysis and AI Predictions”. **B** Predictions matches the in situ gene expression profile with the ICCA like area showing upregulation of biliary/cholangiocytic genes (*EPCAM, HNF1B* and *KRT7*) and downregulation of hepatocytic genes (*ALB, FABP1* and *APOB*). Raw data are available online as described in the “Data Availability” Section.

To gain further insights into the in situ relationships between the models prediction and the underlying biology, we performed spatial transcriptomics on tissue sections obtained from formalin-fixed, paraffin embedded blocks of 6 randomly selected cHCC-CCA cases. We then applied our model on the corresponding WSI and matched the prediction heatmaps with the gene expression profiling data. We investigated, within each case, the differences between the 100 image tiles most highly associated with a prediction of ICCA and the 100 tiles most highly associated with a prediction of HCC. We observed that the model’s predictions matched the underlying in situ gene expression profiles of the tumors. For Sample #A4 (Fig. 4A and B), areas predicted as ICCA-like were indeed associated with increased expression of genes related to cholangiocyte differentiation (e.g *EPCAM*, *HNF1B* and *KRT7*) and decreased expression of well-known hepatocytic markers (*ALB*, *FABP1* and *APOB*) (Fig. 4B and Supplementary Data 1). Similar findings were obtained for 2 additional cases (#A1 and #A2), while the results for remaining samples were less clear with few significantly dysregulated genes (#A3, #A5 and #A6) (Supplementary Data 1), possibly due to the constraints of performing spatial transcriptomics analyses on formalin-fixed paraffin-embedded material.

## Discussion

In summary, our study shows that AI-based reclassification of cHCC-CCA into one of the “pure” HCC or CCA categories could improve prognostication, which is critical given the therapeutic implications, and also help to determine if a given cHCC-CCA tumor is genetically more similar to HCC or ICCA. A diagnosis of cHCC-CCA remains a formidable challenge for physicians and little or no evidence is available to guide the treatment options for the patients. Hence, oncologists often recommend treatment according to HCC or CCA therapeutic strategies, but the responses are often poor and their outcome dire^3,15^. Recent large scale molecular studies of cHCC-CCA have failed to demonstrate any specific genetic alterations, and most cases have a similar gene expression profile to that of HCC or ICCA^3,4^. This reclassification could be performed by genetic profiling, however these approaches are not universally available and are lengthy and costly. By definition, routine histopathological slides are available for every single one of these patients as histopathological evaluation is needed to make a diagnosis of cHCC-CCA in the first place.

Here, we have shown that an AI system can make a clear call for either HCC or ICCA. Reclassifying tumors as ICCA could be clinically useful as some of the associated alterations, including *FGFR2* fusions and *BRAF* and *IDH* mutations, can be targeted by specific drugs. Measurable antitumor activity has indeed been reported with pemigatinib, futibatinib (*FGFR* inihbitors), ivosidenib (*IDH1* inhibitor) or neratinib (pan-HER tyrosine kinase inhibitor)^16–18^. The standard of care for patients with advanced disease is also different between HCC (atezolizumab plus bevacizumab or durvalumab plus tremelimumab) and ICCA (durvalumab plus chemotherapy), and prospective clinical trials, although very challenging to carry out, are needed to determine if patients with cHCC-CCA may benefit from our reclassification approach to be allocated the systemic treatment that fits with the predicted class.

A limitation of our study is that by the nature of this problem, there is no ground truth for our proposed reclassification. We rely on a combination of clinical and genomic markers to demonstrate the plausibility and utility of our proposed reclassification scheme. We further observe that as for any biomarker, model predictions close to the decision cutoff are associated with a higher ambiguity. For such cases falling in the mid-range of the AI score, pathologists could integrate other factors such as clinical probability or imaging results to enhance the prediction confidence. Our software is currently suitable for research use only, and regulatory approvals will be needed to ensure its reliability and efficacy. The sharing of our source code however encourages further development and application by the wider research community.

The next step for the implementation of such models will be their validation on biopsies, as they are the only type of samples that can be obtained before surgery (resection or transplantation) or in patients with advanced disease not amenable for curative therapies. It may be challenging as biopsy is rarely performed due to the existence of non-invasive HCC diagnostic criteria. There is however a renewed interest in biopsy, in particular in the context of clinical trials, and this validation process could be undertaken in the near future. This could also be an opportunity to determine whether the addition of biological features (AFP/CA 19-9) or radiological findings may improve the classification.

In conclusion, our study demonstrates that AI may be useful for tumors that do not fit into common nosological frameworks. Developing evidence-based guidelines for such rare and challenging entities is indeed difficult (if not impossible). Our method could be applied to other cancer subtypes with mixed or biphenotypic differentiation that present a therapeutic challenge, such as combined adenocarcinomas / neuroendocrine tumors or adenosquamous carcinomas. We also believe that the combination of deep-learning heatmaps with spatial transcriptomics is a useful approach to provide insights into the molecular profile of highly predictive areas, and thus demonstrates that AI can be used as a tool for understanding tumor tissue in a research context.

## Methods

### Ethics statement

This study reports a retrospective analysis of tissue samples of archival tissue of primary liver tumors which was collected in a multicentric way. The protocol was approved by the review board of Université Paris Est Creteil, France (ID n° APHP22012), conducted in accordance with the Declaration of Helsinki and the legislations of each participating center. In this international multicentric cohort informed consents were obtained from patients when required by local regulations. Centers with informed written consent obtained: Hamburg, Barcelona, Mondor, Chinese University of Hong Kong, Beaujon, Paul Brousse. Centers with waiver of consent after IRB approval: University of Texas Southwestern, Stanford, Aachen, Pitié-Salpêtrière, Michigan University, Chennai, Rouen, Saint Antoine, Lille, Angers, Milano, Amiens, Hong Kong, Poitiers, St Louis University, Seoul National University College of Medicine, Prince of Songkhla, Montpellier, Brest, Reims, Yale School of Medicine, Bachmai Hospital, Mayo Clinic Rochester, Regensburg.

### Patients and samples

Slides used for training (pure HCC and iCCA) were obtained from the archives of five pathology departments. Inclusion criteria were as follows: (1) patients with HCC or ICCA treated by surgical resection, (2) lack of preoperative antitumor treatment and (3) available WSI and baseline clinical, biological and pathological features. They were scanned using a Hamamatsu Nanozoomer S360 (ndpi encoding format) or a Leica Aperio (svs encoding format) scanning device. For the validation cohort, we used TCGA-LIHC and TCGA-CHOL cohorts (HCC *n* = 333 and ICCA *n* = 27). Slides of cHCC-CCA were obtained from European (*n* = 18), American (*n* = 6) and Asian (*n* = 6) liver centers. Inclusion criteria were: patients treated by surgical resection or liver transplantation, diagnosis of cHCC-CCA as defined by the World Health Organization and available histological slides and baseline clinical data.

### Pathological reviewing

For all cases, an expert liver pathologist (JC) reviewed the cHCC-CCA histological slides and quantified each contingent (HCC, ICCA, and intermediate/equivocal). To compare the AI model’s prediction with a conventional morphological reclassification, cHCC-CCA cases were reclassified as HCC if the HCC contingent was more abundant that the ICCA contingent or as ICCA if the ICCA was more abundant that the HCC contingent.

### Development and validation of the deep learning model

Processing of WSIs was performed according to a pre-defined protocol^19^. Digitized WSI were preprocessed by tessellation into non-overlapping small patches of size $224\times 224\times 3$ pixels at an edge length of 256 µm. Background and blurry tiles were removed in order to provide the deep learning model with clean and informative input. As described before, the Canny Edge detector module with a threshold of 2 from OpenCV was used^20^. The primary analysis was carried out using the raw image tiles, and was repeated after color-normalization with the Macenko method to investigate potential batch effects^21^. Then, we used our previously published pipeline “Marugoto” for supervised Deep Learning^12,13^. The pipeline consists of a feature extraction module which transforms each tile into a feature vector of size $1\times 2048$. Feature vectors of all tiles for each slide are subsequenctly processed by an aggregation module which outputs a single score for a given WSI. As the feature extraction module, we used a resnet50 which was pre-trained in a self-supervised way with the RetCCL method in a previous study^11^. The pretraining included TCGA image data but no labels. As the aggregation module, we used a custom-built attention-based multiple instance learning (attMIL)^22^. AttMIL incorporates an attention mechanism that involves two fully connected layers that compute an attention score for each tile, resulting in a bag-level feature vector obtained by scaling the embeddings of each tile using the softmax of its attention score, and adding them up^22^. This bag-level feature vector is then transformed into a final classification through another fully connected layer. To train the model on multiple patients in each batch, a subset of tiles from each patient is considered sufficient, and in each epoch, the tiles are re-sampled^20,22^. We trained the model for 100 epochs with a patient of 16 for an early-stopping callback. We used a batch size of 64 for the training subset and defined a fixed bag size of 512. For every training epoch, the instances for each bag have been randomly re-sampled. We used all the tiles of the WSIs for prediction in the test and validation cohorts, with a batch size of 1. In order to evaluate the model’s internal performance, we performed a 5-fold cross-validation at the level of patients.

### Identification of molecular alterations

Tumor areas were first macro-dissected from formalin-fixed, paraffin embedded tissue blocks, and mRNA and DNA extractions were performed using the Maxwell RSC Plus DNA FFPE Kit IVD and the Maxwell RSC RNA FFPE Kit IVD (Promega, France). They were further quantified using a Qubit fluorimeter in combination with the Qubit™ dsDNA HS Assay Kit and Qubit™ RNA HS Assay Kit (ThermoFisher Scientific). RNA was reverse transcribed to cDNA using SuperScript IV VILO Master Mix (ThermoFisher Scientific). The Oncomine Comprehensive Library Assay v3C was used to amplify 50 nanograms of DNA and RNA (as measured by fluorimetry). Amplicons were digested, barcoded and amplified with the Ion Ampliseq Library and Ion Xpress barcode adapter kits (ThermoFisher Scientific). After quantification, 50 pM of each library were multiplexed and clonally amplified on ion-sphere particles using a Ion Chef instrument (ThermoFisher Scientific). The ISP templates were loaded onto an Ion-540 chip and sequenced using an Ion S5 device and the Ion 540™ Kit–Chef. The Ion Reporter Software was used to assess performance and analyze sequencing data using specific stringent filters (allele frequency between 5 and 90%; only exonic location, read depth >300X).

### Spatial transcriptomics

Samples were first screened for RNA quality (DV200 scores > 50%, Tapestation), as recommended on the Visium Tissue Preparation Guide (10X Genomics). Visium spatial gene expression slides and reagents kits were used according to manufacturer instructions.

Five-micrometer thick tissue sections were cut from the FFPE tumor block and placed within the fiducial frames (*n* = 4) of the FFPE Visium Spatial Gene Expression Slide. Each capture areas has ~5000 gene expression spots that include a partial read 1 sequencing primer (Illumina TruSeq Read 1), 16 nt spatial barcode, a 12 nt unique molecular identifier (UMI) and a 30 nt poly(dT) sequence (captures ligation product). Spots provide a resolution of ~5–10 cells. Slides were deparaffinized and stained. They were further coverslipped and scanned at 40X resolution using a Hamamatsu S360 scanning device. Coverslips were removed, and a decrosslinking step was performed.

Probes were hybridized using the Visium Hybridization Mix. After a post-hybridization wash (FFPE Post-Hyb Wash and SSC Buffers), a ligase is added to seal the junction between the probe pairs that have hybridized to RNA, forming a ligation product. The ligation products were released from the tissue section upon RNase treatment and permeabilization, and further captured on the slide. Probes were extended by the addition of UMI, partial read 1 and spatial barcodes. We then obtained spatially barcoded products for library preparation.

A qPCR was performed to determine the cycle numbers, and the ligated and spatially barcoded products underwent indexing via Sample Index PCR.Sequencing of libraries was performed on a NextSeq 2000 instrument with a P3 flow cell (100 cycles, Illumina, CA, USA).

### Survival analyses

Statistical analysis and visualization were performed using R software version 4.0.2 (R Foundation for Statistical Computing, Vienna, Austria. https://www.R-project.org) and Bioconductor packages (version 3.4). Overall survival was defined by the interval between surgical resection/liver transplantation and death or last follow-up. Survival curves were represented using the Kaplan-Meier method compared with log-rank statistics. Univariate analysis was performed using the Cox proportional-hazards regression model with variables with a *P*-value < 0.05 selected for multivariate analysis. All tests were two-tailed and a *P*-value < 0.05 was considered significant. For patients treated by surgical resection, inclusion criteria were lack of pre-operative treatment, lack of metastatic or macroscopic residual disease at the time of surgery, and uninodular tumors. For liver transplantation, all patients with available clinical follow-up were included.

### Statistics, reproducibility and other tools

Measurements were taken from distinct samples, i.e., the same sample was never measured repeatedly. For supervised classification experiments, the primary endpoint was the area under the receiver operating characteristic curve (AUROC) with 95% confidence intervals obtained by 1000x bootstrapping. The MI-CLAIM checklist is provided in Supplemental Table 7. In accordance with the COPE (Committee on Publication Ethics) position statement of 13 February 2023 (https://publicationethics.org/cope-position-statements/ai-author), the authors hereby disclose the use of the following artificial intelligence models during the writing of this article. GPT-4 (OpenAI) for checking spelling and grammar.

### Reporting summary

Further information on research design is available in the Nature Portfolio Reporting Summary linked to this article.

### Supplementary information

Supplementary Information

Peer review file

Description of Additional Supplementary Files

Supplementary Data 1

Reporting Summary

### Source data

Source Data

## Data Availability

Some of the data that support the findings of this study are publicly available, and some are proprietary datasets provided for this analysis under collaboration agreements. All data (including histological images) from the TCGA database are available at https://portal.gdc.cancer.gov. Raw sequencing data for the proprietary cohorts have been uploaded to the European Nucleotide Archive (ENA) (accession number PRJEB62487). All other histopathology image data with accompanying metadata are under controlled access according to the local ethical guidelines and can only be requested directly from the respective study groups that independently manage data access for their study cohorts. The central data collection was managed by JC to whom sharing requests can be directed and will be responded to within 4 weeks. Source data for figures are provided with this paper.

## Code availability

The data was analyzed using custom-developed open-source software. Our deep learning methods use Python with h5py v3.6, numpy v1.22, openpyxl v3.0, pandas v1.4, torch v1.8, fastai v2.5, fire v0.4. The transcriptomics analysis and statistical analysis methods use R v4.1.2, Seurat v4.1.1 for differential gene expression analysis, Seurat v4.3.0 for visualization, glmGamPoi v1.6.0, dplyr v1.0.10, crayon v1.5.2, ggplot2 v3.4.0, gridExtra v2.3, MAST v1.20.0, ggrepel v0.9.2, readxl v1.4.3, survminer v0.4.9, rms v6.4-1, survival v3.5-5, ComplexHeatmap v2.13.1, irr v0.84.1, vcd v1.4-11. All source codes are publicly available: https://github.com/KatherLab/preprocessing-ng for WSI tessellation, https://github.com/KatherLab/preProcessing for color-normalization and https://github.com/KatherLab/marugoto for model training and deployment and https://github.com/qinghezeng/ST_cHCC-CCA for spatial transcriptomics analysis.
