# Supplementary material for: Deep learning-based phenotyping reclassifies combined hepatocellular-cholangiocarcinoma
Source: Nat Commun. 2023 Dec 14;14:8290. doi: 10.1038/s41467-023-43749-3 (PMC10719304; doi:10.1038/s41467-023-43749-3)
Supplement: Supplementary file 1 — Supplementary Information [file 41467_2023_43749_MOESM1_ESM.docx]

# Supplemental Tables ​​

| **Variable** | **Available data** | **n(%)** |
| --- | --- | --- |
| **Gender** (Male) | 424 | 356 (84) |
| **Age>60 yrs** | 424 | 248 (58) |
| **Etiology** (Alcohol) | 403 | 116 (29) |
| **Etiology** (HCV) | 403 | 100 (25) |
| **Etiology** (HBV) | 403 | 109 (27) |
| **Etiology** (NASH) | 403 | 67 (17) |
| **Etiology** (Undetermined) | 403 | 48 (12) |
| **BCLC Disease Stage** | 419 | 282 (67)/137 (33) |
| **Treatment before intervention** | 420 | 43 (10) |
| **Preoperative AFP** (>20 ng/mL) | 350 | 148 (42) |
| **Largest nodule diameter** (≥50 mm) | 418 | 193 (46) |
| **Multinodularity** | 419 | 84 (20) |
| **Macrovascular invasion** | 423 | 91 (21) |
| **Microvascular invasion** | 423 | 216 (51) |
| **Surgical margins (R1)** | 423 | 55 (13) |
| **Cirrhosis** | 403 | 160 (40) |

**Supplemental Table 1. Clinical, biological and pathological features of patients and tumors from the HCC cohort (n=424).**

| **Variable** | **Available data** | **n(%)** |
| --- | --- | --- |
| **Gender** (Male) | 167 | 100 (60) |
| **Age>60 yrs** | 167 | 78 (47) |
| **Etiology** (Alcohol) | 145 | 8 (5) |
| **Etiology** (HCV) | 145 | 11 (8) |
| **Etiology** (HBV) | 145 | 13 (9) |
| **Etiology** (NASH) | 145 | 24 (16) |
| **Etiology** (Undetermined) | 145 | 89 (61) |
| **Largest nodule diameter** (≥50 mm) | 167 | 41 (24) |
| **Multinodularity** | 167 | 22 13) |
| **Microvascular invasion** | 141 | 22 (16) |
| **Surgical margins (R1)** | 141 | 18 (13) |
| **Cirrhosis** | 164 | 20 (12) |

**Supplemental Table 2. Clinical, biological and pathological features of patients and tumors from the ICCA cohort (n=167).**

| **Variable** | **Available data** | **n(%)** |
| --- | --- | --- |
| **Gender** (Male) | 405 | 313 (77) |
| **Age>60 yrs** | 396 | 255 (64) |
| **Etiology** (Alcohol) | 357 | 100 (28) |
| **Etiology** (HCV) | 357 | 87 (24) |
| **Etiology** (HBV) | 357 | 55 (15) |
| **Etiology** (NASH) | 357 | 69 (19) |
| **Etiology** (Undetermined) | 357 | 75 (21) |
| **Type of treatment (Resection/Transplantation)** | 398 | 303 (76)/95 (24) |
| **Treatment before intervention** | 325 | 70 (21) |
| **Preoperative AFP** (>20 ng/mL) | 193 | 123 (64) |
| **Largest nodule diameter** (≥50 mm) | 391 | 120 (31) |
| **Multinodularity** | 358 | 111 (31) |
| **Macrovascular invasion** | 362 | 33 (9) |
| **Microvascular invasion** | 371 | 190 (51) |
| **Surgical margins (R1)** | 330 | 38 (11) |

# Supplemental Table 3. Clinical, biological and pathological features of patients and tumors from the cHCC-CCA cohort.

|  | | | | | |  |  |  |  |
| --- | --- | --- | --- | --- | --- | --- | --- | --- | --- |
|  |  | **Univariate** | | | | **Multivariate** | | | |
| **Variable** | **Available data** | **Hazard ratio** | **95% CI (lower)** | **95% CI (upper)** | **P-value** | **Hazard ratio** | **95% CI (lower)** | **95% CI (upper)** | **P-value** |
| ***Overall cohort (n = 126; deaths n = 53)*** |  |  |  |  |  |  |  |  |  |
| **Gender** (Male) | 126 | 2.7179 | 1.2802 | 5.7705 | **0.0092** | 3.149 | 1.4152 | 7.006 | **0.00494** |
| **Age at intervention** | 118 | 0.9951 | 0.9705 | 1.0203 | 0.7004 | - | - | - | - |
| **Etiology** (Alcohol) | 109 | 1.3373 | 0.7309 | 2.4468 | 0.3457 | - | - | - | - |
| **Etiology** (HCV) | 110 | 1.0385 | 0.4832 | 2.2318 | 0.9229 | - | - | - | - |
| **Etiology** (HBV) | 109 | 0.9253 | 0.4701 | 1.8212 | 0.8222 | - | - | - | - |
| **Etiology** (NASH) | 109 | 1.4690 | 0.7086 | 3.0453 | 0.3012 | - | - | - | - |
| **Etiology** (Undertermined) | 108 | 0.7101 | 0.3681 | 1.3699 | 0.3071 | - | - | - | - |
| **Largest nodule diameter** (≥50 mm) | 126 | 1.7798 | 1.0264 | 3.0862 | **0.0401** | 1.346 | 0.7584 | 2.387 | 0.31025 |
| **Preoperative AFP** (>20 ng/mL) | 67 | 1.1165 | 0.5516 | 2.2600 | 0.7593 | - | - | - | - |
| **Surgical margins** (R1) | 126 | 1.5736 | 0.7843 | 3.1571 | 0.2019 | - | - | - | - |
| **Microvascular invasion** | 123 | 3.4203 | 1.8638 | 6.2765 | **0.0001** | 3.058 | 1.645 | 5.684 | **0.00041** |
| **Cirrhosis** | 104 | 1.1540 | 0.6415 | 2.0760 | 0.6326 | - | - | - | - |
| **AI Prediction** (ICCA) | 126 | 1.7583 | 0.9843 | 3.1409 | **0.0566** | 1.526 | 0.8472 | 2.748 | 0.15927 |

**Supplementary Table 4. Univariate and Multivariate Cox regression analysis for overall survival in patients with cHCC-CCA treated by surgical resection.** CI= Confidence Interval. Statistical tests were two-sided and not adjusted for multiple testing.

|  | | | | | | |  |  |  |
| --- | --- | --- | --- | --- | --- | --- | --- | --- | --- |
|  |  | **Univariate** | | | | **Multivariate** | | | |
| **Variable** | **Available data** | **Hazard ratio** | **95% CI (lower)** | **95% CI (upper)** | **P-value** | **Hazard ratio** | **95% CI (lower)** | **95% CI (upper)** | **P-value** |
| ***Overall cohort (n = 87; deaths n = 24)*** |  |  |  |  |  |  |  |  |  |
| **Gender** (Male) | 87 | 1.7056 | 0.5072 | 5.7353 | 0.3882 | - | - | - | - |
| **Age at intervention** | 78 | 1.0079 | 0.9534 | 1.0656 | 0.7811 | - | - | - | - |
| **Etiology** (Alcohol) | 83 | 0.4512 | 0.1974 | 1.0313 | **0.0592** | 0.5633 | 0.2357 | 1.346 | 0.1967 |
| **Etiology** (HCV) | 83 | 1.2467 | 0.1681 | 9.2459 | 0.8292 | - | - | - | - |
| **Etiology** (HBV) | 83 | 1.3425 | 0.5949 | 3.0294 | 0.4781 | - | - | - | - |
| **Etiology** (NASH) | 83 | 0.9547 | 0.3248 | 2.8063 | 0.9328 | - | - | - | - |
| **Treatment before intervention** | 77 | 0.9010 | 0.3974 | 2.0429 | 0.8029 | - | - | - | - |
| **Largest nodule diameter** (≥50 mm) | 86 | 3.9126 | 1.7258 | 8.8705 | **0.0011** | 2.3511 | 0.9605 | 5.755 | 0.0612 |
| **Multinodularity** | 83 | 1.2387 | 0.4181 | 3.6696 | 0.6993 | - | - | - | - |
| **Preoperative AFP** (>20 ng/mL) | 39 | 0.8478 | 0.1642 | 4.3774 | 0.8437 | - | - | - | - |
| **Microvascular invasion** | 84 | 4.2188 | 1.7846 | 9.9733 | **0.0010** | 2.72 | 1.0799 | 6.851 | **0.0338** |
| **AI Prediction** (ICCA) | 87 | 2.6937 | 1.1499 | 6.3098 | **0.0225** | 2.7651 | 1.1223 | 6.812 | **0.027** |

**Supplementary Table 5. Univariate and Multivariate Cox regression analysis for overall survival in patients with cHCC-CCA treated by liver transplantation.** Statistical tests were two-sided and not adjusted for multiple testing.

|  | **Resection** (n=126) | | **Transplantation** (n=87) | |  |
| --- | --- | --- | --- | --- | --- |
|  | **Available data** | **n (%)** | **Available data** | **n (%)** | **P-value** |
| **Gender** (Male) | 126 | 89 (70.6) | 87 | 73 (83.9) | **0.039** |
| **Age at surgery** (mean (SD)) | 118 | 62.47 (11.31) | 78 | 59.90 (7.19) | 0.076 |
| **Etiology** |  |  |  |  |  |
| *Alcohol* | 109 | 30 (27.5) | 83 | 45 (54.2) | **<0.001** |
| *HBV* | 110 | 20 (18.2) | 83 | 3 (3.6) | **0.004** |
| *HCV* | 109 | 24 (22.0) | 83 | 28 (33.7) | 0.1 |
| *NASH* | 109 | 16 (14.7) | 83 | 16 (19.3) | 0.515 |
| *Undetermined* | 108 | 35 (32.4) | 82 | 4 (4.9) | **<0.001** |
| **Largest nodule diameter** (≥50mm) | 126 | 50 (39.7) | 86 | 17 (19.8) | **0.004** |
| **Cirrhosis** | 104 | 42 (40.4) | 83 | 80 (96.4) | **<0.001** |
| **Multinodularity** | 124 | 0 (0.0) | 83 | 63 (75.9) | **<0.001** |
| **Preoperative AFP** >20ng/ml | 67 | 22 (32.8) | 39 | 13 (33.3) | 1 |
| **Microvascular invasion** | 123 | 65 (52.8) | 84 | 32 (38.1) | 0.052 |
| **AI-class prediction** (ICCA) | 126 | 79 (62.7) | 87 | 40 (46.0) | **0.023** |

**Supplementary Table 6. Differences in baseline clinicopathological features between resected and transplanted patients.** SD: Standard Deviation

| Before paper submission | | | |
| --- | --- | --- | --- |
| Study design (Part 1) | Completed: page number | | Notes if not completed |
| The clinical problem in which the model will be employed is clearly detailed in the paper. | X |  |  |
| The research question is clearly stated. | X |  |  |
| The characteristics of the cohorts (training and test sets) are detailed in the text. | X |  |  |
| The cohorts (training and test sets) are shown to be representative of real-world clinical settings. | X |  |  |
| The state-of-the-art solution used as a baseline for comparison has been identified and detailed. | X |  |  |
| Data and optimization (Parts 2, 3) | Completed: page number | |  |
| The origin of the data is described and the original format is detailed in the paper. | X |  |  |
| Transformations of the data before it is applied to the proposed model are described. | X |  |  |
| The independence between training and test sets has been proven in the paper. | X |  |  |
| Details on the models that were evaluated and the code developed to select the best model are provided. | X |  |  |
| Is the input data type structured or unstructured? | ☐ Structured X Unstructured | | |
| Model performance (Part 4) | Completed: page number | |  |
| The primary metric selected to evaluate algorithm performance (e.g., AUC, F-score, etc.), including the justification for selection, has been clearly stated. | X |  |  |
| The primary metric selected to evaluate the clinical utility of the model (e.g., PPV, NNT, etc.), including the justification for selection, has been clearly stated. | X |  |  |
| The performance comparison between baseline and proposed model is presented with the appropriate statistical significance. | X |  |  |
| Model examination (Part 5) | Completed: page number | |  |
| Examination technique 1^a^ | X |  |  |
| Examination technique 2^a^ | X |  |  |
| A discussion of the relevance of the examination results with respect to model/algorithm performance is presented. | X |  |  |
| A discussion of the feasibility and significance of model interpretability at the case level if examination methods are uninterpretable is presented. | X |  |  |
| A discussion of the reliability and robustness of the model as the underlying data distribution shifts is included. | X |  |  |
| Reproducibility (Part 6): choose appropriate tier of transparency | | |  |
| Tier 1: complete sharing of the code | | X |  |
| Tier 2: allow a third party to evaluate the code for accuracy/fairness; share the results of this evaluation | | ☐ |  |
| Tier 3: release of a virtual machine (binary) for running the code on new data without sharing its details | | ☐ |  |
| Tier 4: no sharing | | ☐ |  |

**Supplementary Table 7. MI-CLAIM checklist. PPV, positive predictive value; NNT, numbers needed to treat.**

# Supplemental Figures

**
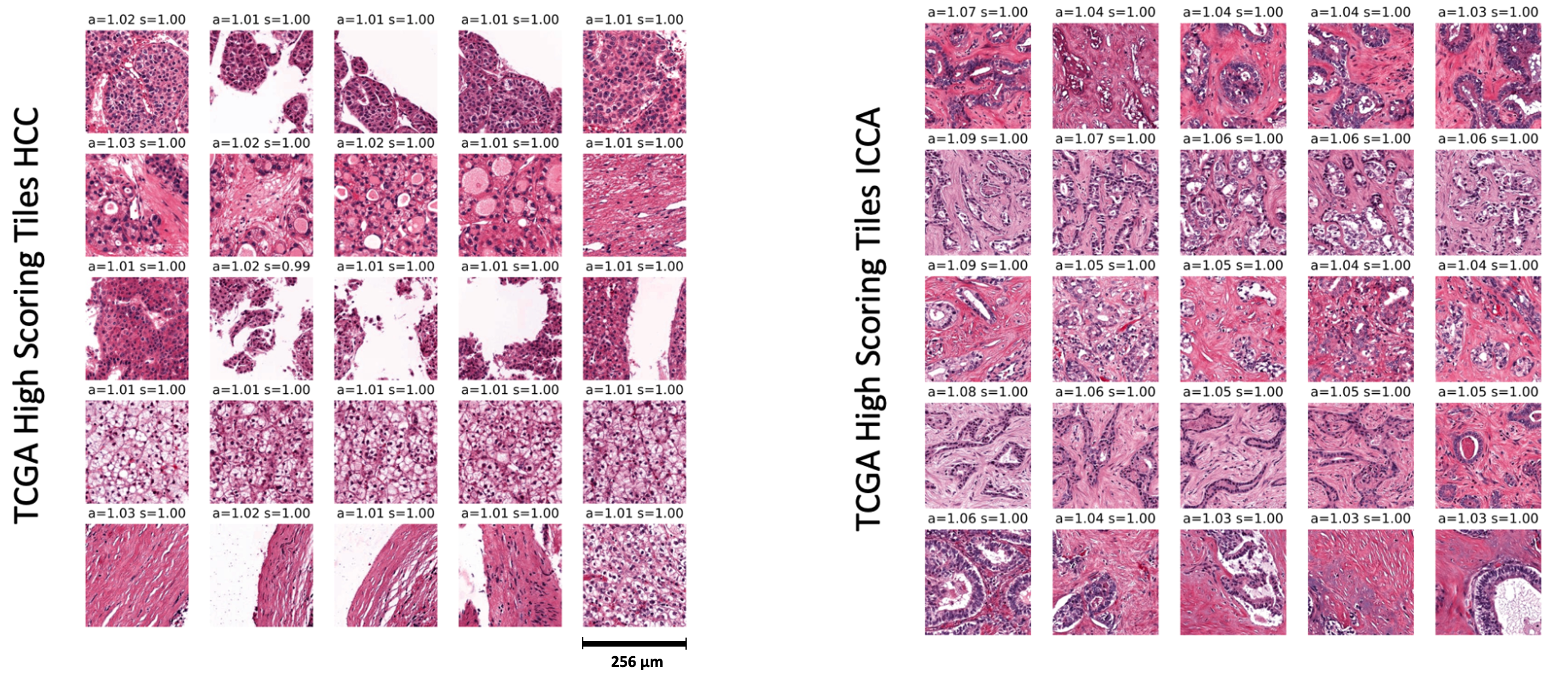
**

**Supplemental Figure 1. Tiles with a high predictive value for HCC or ICCA in the TCGA cohort.** Tiles associated with a diagnosis of HCC (left panel) mainly show large neoplastic cells with an eosinophilic or clear cytoplasm, arranged in a compact, pseudoglandular or trabecular architecture. Tiles associated with a diagnosis of ICCA (right panel) display small ductules or glands embedded in a dense fibrous stroma. a= attention score and s=prediction score.


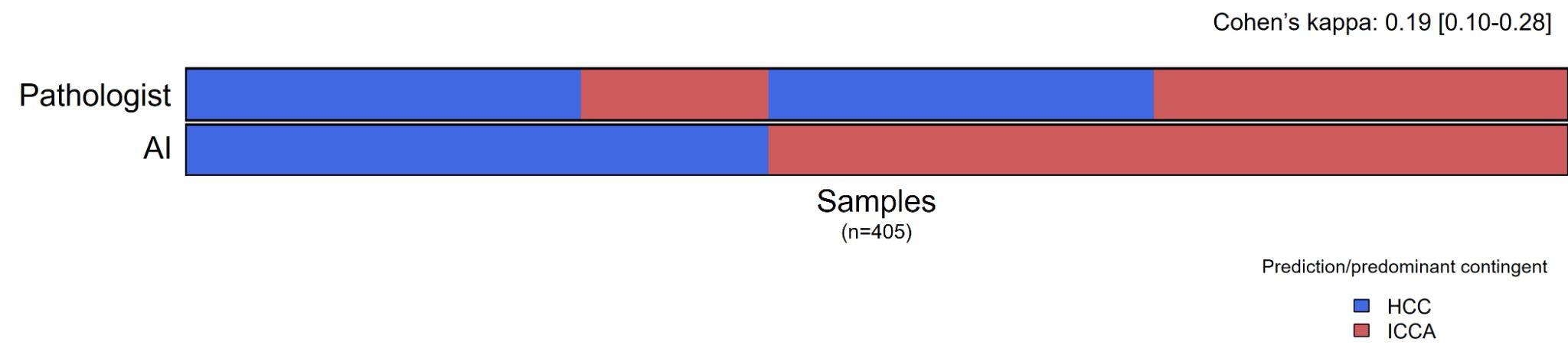


**
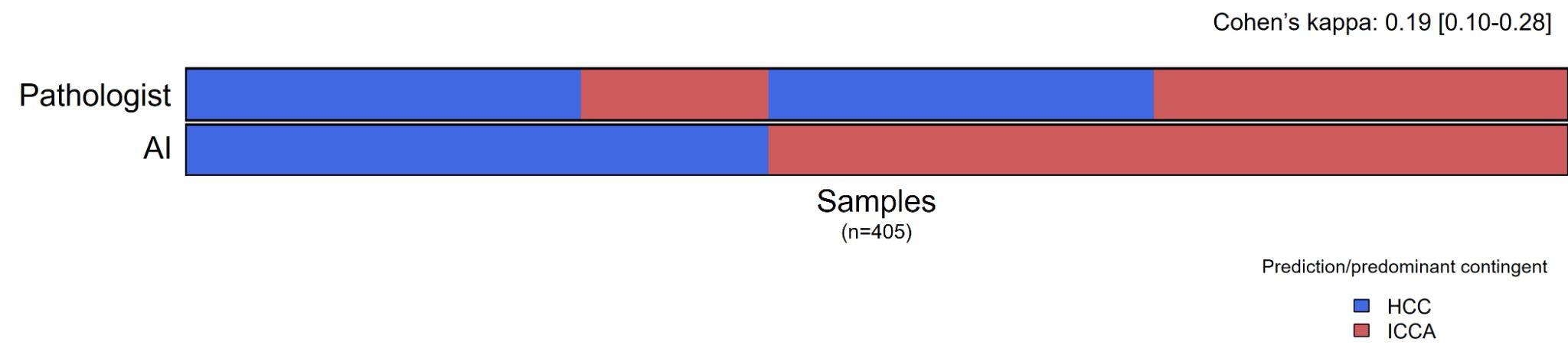
**

**Supplemental Figure 2. Concordance between pathologist’s and AI’s classifications.** Each sample is one case of cHCC-ICCA. AI predictions and the pathological assessment only showed a slight concordance (Cohen’s kappa 0.19). The pathologist assessed which component was more prevalent in the slide HCC or ICCA. The AI model, in contrast, was trained on pure HCCs and pure ICCAs and made a prediction on the combined tumors.


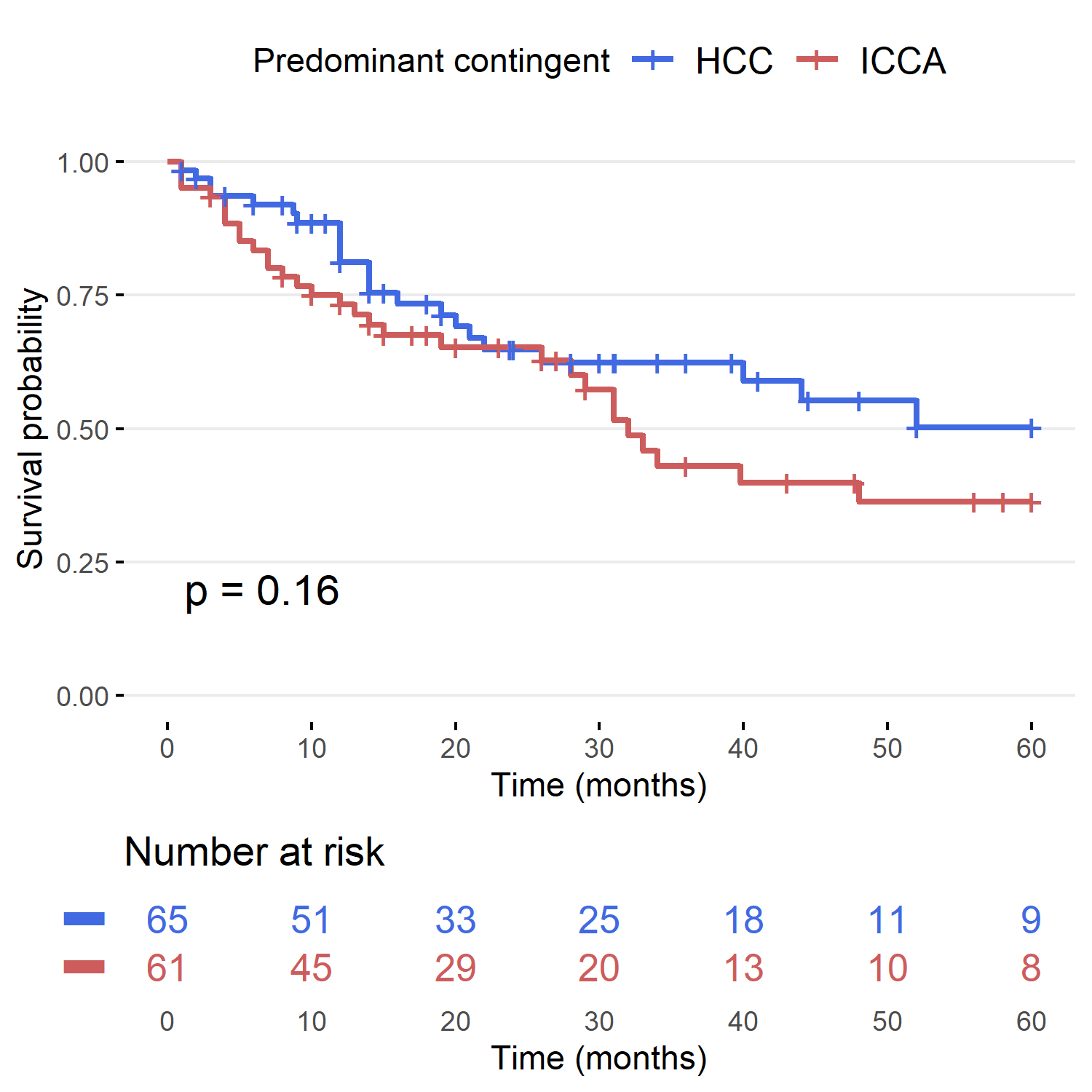


**Supplemental Figure 3. Overall survival after surgical resection in patients with cHCC-CCA, manually reclassified by a pathologist according to the histologically predominant contingent.** There is no significant difference among the two groups of patients (predominant HCC contingent and ICCA predominant contingent. The p-value was calculated with the log rank test.


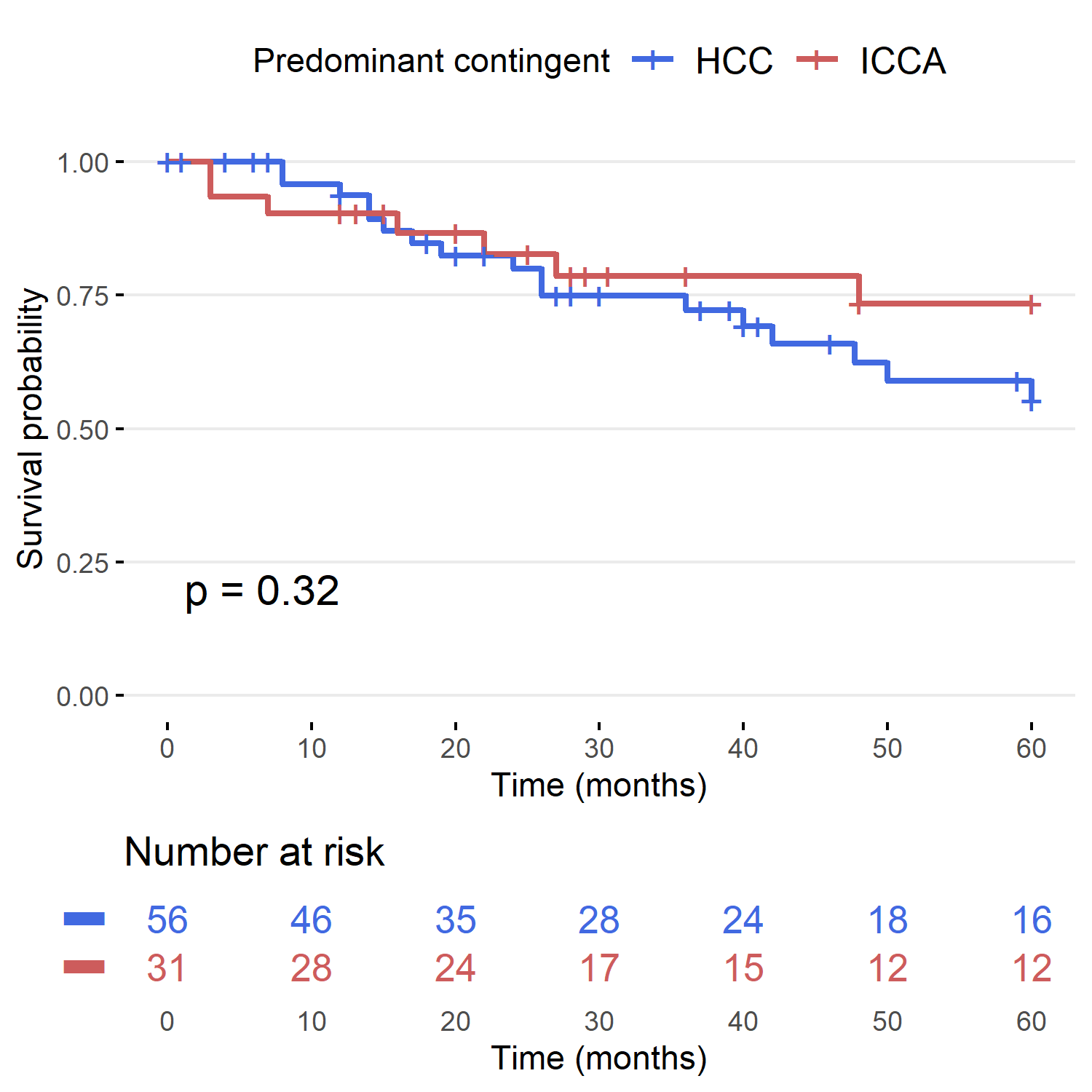


**Supplemental Figure 4. Overall survival after liver transplantation in patients with cHCC-CCA, manually reclassified by a pathologist according to the histologically predominant contingent.** There is no significant difference among the two groups of patients (predominant HCC contingent and ICCA predominant contingent. The p-value was calculated with the log rank test.
