# Supplementary material for: Deep learning-based phenotyping reclassifies combined hepatocellular-cholangiocarcinoma
Source: Nat Commun. 2023 Dec 14;14:8290. doi: 10.1038/s41467-023-43749-3 (PMC10719304; doi:10.1038/s41467-023-43749-3)
Supplement: Supplementary file 3 — Description of Additional Supplementary Files [file 41467_2023_43749_MOESM3_ESM.pdf]

Description of Additional Supplementary Files

Supplementary Data 1: For all six cases analyzed with in situ gene expression profiles available, the differential gene expression between the 100 ICCA-high prediction and 100 HCC-high prediction pat ches for each case is listed here.
