# Supplementary material for: Deep learning-based phenotyping reclassifies combined hepatocellular-cholangiocarcinoma
Source: Nat Commun. 2023 Dec 14;14:8290. doi: 10.1038/s41467-023-43749-3 (PMC10719304; doi:10.1038/s41467-023-43749-3)
Supplement: Supplementary file 5 — Reporting Summary [file 41467_2023_43749_MOESM5_ESM.pdf]

nature portfolio

Corresponding author(s): JN Kather

Last updated by author(s): 2023-NOV-5

Reporting Summary

Nature Portfolio wishes to improve the reproducibility of the work that we publish. This form provides structure for consistency and transparency in reporting. For further information on Nature Portfolio policies, see our Editorial Policies and the Editorial Policy Checklist.

Statistics

For all statistical analyses, confirm that the following items are present in the figure legend, table legend, main text, or Methods section.

n/a	Confirmed	
☐	☒	The exact sample size (*n*) for each experimental group/condition, given as a discrete number and unit of measurement
☐	☒	A statement on whether measurements were taken from distinct samples or whether the same sample was measured repeatedly
☐	☒	The statistical test(s) used AND whether they are one- or two-sided<br>*Only common tests should be described solely by name; describe more complex techniques in the Methods section.*
☐	☒	A description of all covariates tested
☐	☒	A description of any assumptions or corrections, such as tests of normality and adjustment for multiple comparisons
☐	☒	A full description of the statistical parameters including central tendency (e.g. means) or other basic estimates (e.g. regression coefficient) AND variation (e.g. standard deviation) or associated estimates of uncertainty (e.g. confidence intervals)
☐	☒	For null hypothesis testing, the test statistic (e.g. *F*, *t*, *r*) with confidence intervals, effect sizes, degrees of freedom and *P* value noted<br>*Give P values as exact values whenever suitable.*
☒	☐	For Bayesian analysis, information on the choice of priors and Markov chain Monte Carlo settings
☒	☐	For hierarchical and complex designs, identification of the appropriate level for tests and full reporting of outcomes
☒	☐	Estimates of effect sizes (e.g. Cohen's *d*, Pearson's *r*), indicating how they were calculated

Our web collection on statistics for biologists contains articles on many of the points above.

Software and code

Policy information about availability of computer code

Data collection	No specific software was used for data collection.
Data analysis	The data was analyzed using custom-developed open-source software. Our deep learning methods use Python with h5py v3.6, numpy v1.22, openpyxl v3.0, pandas v1.4, torch v1.8, fastai v2.5, fire v0.4. The transcriptomics analysis and statistical analysis methods use R v4.1.2, Seurat v4.1.1 for differential gene expression analysis, Seurat v4.3.0 for visualization, glmGamPoi v1.6.0, dplyr v1.0.10, crayon v1.5.2, ggplot2 v3.4.0, gridExtra v2.3, MAST v1.20.0, ggrepel v0.9.2, readxl v1.4.3, survminer v0.4.9, rms v6.4-1, survival v3.5-5, ComplexHeatmap v2.13.1, irr v0.84.1, vcd v1.4-11. All source codes are publicly available: https://github.com/KatherLab/preprocessing-ng for WSI tessellation, https://github.com/KatherLab/preProcessing for color-normalization and https://github .com/KatherLab/marugoto for model training and deployment and https://github.com/qinghezeng/ ST_cHCC-CCA for spatial transcriptomics analysis.

For manuscripts utilizing custom algorithms or software that are central to the research but not yet described in published literature, software must be made available to editors and reviewers. We strongly encourage code deposition in a community repository (e.g. GitHub). See the Nature Portfolio guidelines for submitting code & software for further information.

Data

Policy information about availability of data

All manuscripts must include a data availability statement. This statement should provide the following information, where applicable:

- Accession codes, unique identifiers, or web links for publicly available datasets
- A description of any restrictions on data availability
- For clinical datasets or third party data, please ensure that the statement adheres to our policy

Some of the data that support the findings of this study are publicly available, and some are proprietary datasets provided for this analysis under collaboration agreements. All data (including histological images) from the TCGA database are available at https://portal.gdc.cancer.gov. Sequencing data for the proprietary cohorts have been uploaded to the European Nucleotide Archive (ENA) (accession number PRJEB62487, available at https://www.ebi.ac.uk/ena/browser/view/PRJEB62487). All other histopathology image data with accompanying metadata are under controlled access according to the local ethical guidelines and can only be requested directly from the respective study groups that independently manage data access for their study cohorts. The central data collection was managed by JC to whom sharing requests can be directed and will be responded to within four weeks. Source data for figures are provided with this paper.

Research involving human participants, their data, or biological material

Policy information about studies with human participants or human data. See also policy information about sex, gender (identity/presentation), and sexual orientation and race, ethnicity and racism.

Reporting on sex and gender	Information about sex was available in the original pathology report. We report this in Suppl. Table 1-3.
Reporting on race, ethnicity, or other socially relevant groupings	This data was not collected.
Population characteristics	This data is available in Suppl. Table 1-3.
Recruitment	This was a retrospective collection of archived tumor tissue of patients with the diagnosis of interest, performed at multiple institutions.
Ethics oversight	The protocol was approved by the review board of Université Paris Est Creteil, France (ID n° APHP22012).

Note that full information on the approval of the study protocol must also be provided in the manuscript.

Field-specific reporting

Please select the one below that is the best fit for your research. If you are not sure, read the appropriate sections before making your selection.

☒ Life sciences ☐ Behavioural & social sciences ☐ Ecological, evolutionary & environmental sciences

For a reference copy of the document with all sections, see nature.com/documents/nr-reporting-summary-flat.pdf

Life sciences study design

All studies must disclose on these points even when the disclosure is negative.

Sample size	There is no formal method to calculate the sample size for histopathology deep learning studies. We planned our sample size to be in the same order of magnitude as the sample size of previous studies, such as Saldanha et al., npj Precision Oncology 2023 (Reference 13). Given that cHCC-CCA is a rare tumor, we obtained a large cohort of N=405 of these tumors.
Data exclusions	All samples with a diagnostic-grade histopathology images (as decided by the pathologist who contributed the samples, before the analysis was run) were included. No data were excluded.
Replication	The experiments were repeated three times and all attempts at reproduction were successful.
Randomization	Not relevant to our study. We did not randomize any patients or samples to any groups. All group assignments of samples were only based on the ground truth diagnosis.
Blinding	All persons who handled any samples did so in a blinded way with respect to the final analysis. No patient groups were allocated in this study.

Reporting for specific materials, systems and methods

We require information from authors about some types of materials, experimental systems and methods used in many studies. Here, indicate whether each material, system or method listed is relevant to your study. If you are not sure if a list item applies to your research, read the appropriate section before selecting a response.

Materials & experimental systems

n/a	Involved in the study
☒	☐ Antibodies
☒	☐ Eukaryotic cell lines
☒	☐ Palaeontology and archaeology
☒	☐ Animals and other organisms
☐	☒ Clinical data
☒	☐ Dual use research of concern
☒	☐ Plants

Methods

n/a	Involved in the study
☒	☐ ChIP-seq
☒	☐ Flow cytometry
☒	☐ MRI-based neuroimaging

Clinical data

Policy information about clinical studies

All manuscripts should comply with the ICMJE guidelines for publication of clinical research and a completed CONSORT checklist must be included with all submissions.

Clinical trial registration: Not applicable, this is a retrospective analysis of archival pathology tissue samples.

Study protocol: Not applicable, not a clinical trial.

Data collection: The data represents a retrospective collection of cHCC-CCA samples from multiple centers. At each center, the local pathologists reviewed archival samples retrospectively and identified a consecutive series of cHCC-CCA which they submitted to the main investigators for analysis. This was done in a completely blinded way with respect to the analysis.

Outcomes: Overall survival was defined by the interval between surgical resection/liver transplantation and death or last follow-up. Survival curves were represented using the Kaplan-Meier method compared with log-rank statistics. Univariate analysis was performed using the Cox proportional-hazards regression model with variables with a P-value < 0.05 selected for multivariate analysis. All tests were two-tailed and a P-value < 0.05 was considered significant. For patients treated by surgical resection, inclusion criteria were lack of pre-operative treatment, lack of metastatic or macroscopic residual disease at the time of surgery, and uninodular tumors. For liver transplantation, all patients with available clinical follow-up were included.
